# 815. Demographics and Outcomes of Rural versus Urban Elderly Burn Patients

**DOI:** 10.1093/jbcr/irag033.254

**Published:** 2026-04-06

**Authors:** Humza Nazir, Jessica Reynolds, Dhaval Bhavsar

**Affiliations:** The University of Kansas Health System Burnett Burn Center, Overland Park, KS; The University of Kansas Medical Center, Kansas City, KS; The University of Kansas Health System, Kansas City, KS

## Abstract

**Introduction:**

Elderly burn patients face disproportionately higher morbidity and mortality even with smaller injuries. It is likely that rural populations face additional struggles like longer commute times and access to specialized care. The purpose of this study was to compare demographic and patient outcomes of rural and urban elderly burn patients at a verified burn center.

**Methods:**

An analysis of data was performed on patients 65 or older admitted between 2000 and 2022. They were classified as rural or urban using Rural–Urban commuting codes from 2020 in relation to patients’ zip codes. A RUCA code of 7 or above was considered rural. Demographics, injury characteristics, comorbidities, procedures, and outcomes were compared. We used t-tests and chi-squares with the significance set to *p*<.05.

**Results:**

A total of 776 patients (mean age 75) were included in the study, with 14.6%(n = 113) from rural areas and 85.4% from urban. Males made up 63.3% of the population with a similar distribution between the rural and urban groups. Majority of the patients were Caucasian (84.4%), though the race distribution varied significantly with no rural African American patients compared to 12.4% in the urban group. Furthermore, 100% of the rural population was non-Hispanic. The mean time to presentation was significantly longer for the rural group (4.69 vs. 2.21). The mean number of OR Procedures was also higher for the rural group (3.43 vs. 2.87). Lastly the payer source varied significantly(*p*=.0045) with the rural population mainly using Medicaid (13.76% vs. 4.98%) and the urban population mainly using private/commercial Insurance (13.84% vs 5.50%). Difference for all other parameters (TBSA, LOS, mortality rate, co-morbidities) were not significant though some (mortality, co-morbidities) reached clinical significance.

**Conclusions:**

In this study of elderly burn patients at a tertiary burn center, rural patients presented later after injury compared to urban patients and required more procedures but showed similar mortality, LOS, and TBSA. Delay in the time to presentation did not adversely impact outcomes.

**Applicability of Research to Practice:**

Recognizing differences in burn care for rural population and the necessity of prevention strategies for elderly rural populations as well as possible guidelines for patient transfer.

**Funding for the study:**

N/A.

